# Efficacy and feasibility of proximal radioulnar derotational osteotomy and internal fixation for the treatment of congenital radioulnar synostosis

**DOI:** 10.1186/s13018-019-1130-0

**Published:** 2019-03-20

**Authors:** Xinjian Pei, Jiuhui Han

**Affiliations:** Department of Pediatric Orthopaedics, The Third Hospital of Hebei Medical University, No. 139 Ziqiang Road, Shijiazhuang, 050051 Hebei China

**Keywords:** Congenital radioulnar synostosis, Derotational osteotomy, Failla classification system, Forearm function

## Abstract

**Background:**

The aim of this study was to assess the feasibility and efficacy of proximal radioulnar derotational osteotomy followed by internal fixation for the treatment of congenital radioulnar synostosis (CRUS).

**Methods:**

Between May 2008 and August 2016, 31 patients (36 forearms) with CRUS who underwent derotational osteotomy at the proximal radioulnar synostosis site were evaluated. There were 20 boys and 11 girls. The mean age at the time of surgery was 4.87 ± 3.06 (range, 2 to 13) years. The forearm was derotated to the goal position (20 degrees of supination to 10 degrees of pronation) using plates for internal fixation and plaster splints for external immobilization. Pre- and postoperative positions of the forearm were recorded; forearm function was evaluated based on the classification system proposed by Failla et al.

**Results:**

The mean follow-up duration was 55.19 ± 27.10 (24 to 123) months. The mean initial pronation deformity was 62.92 ± 7.11 (55 to 80) degrees. The mean correction achieved was 70.86 ± 9.58 (50 to 90) degrees, resulting in a mean final position of 7.94 ± 7.25 degrees of supination (20 degrees of supination to 10 degrees of pronation). Based on the Failla classification system, 2 forearms were rated as good, 30 were rated as fair, and 4 were rated as poor preoperatively. At the final follow-up, 34 forearms were rated as excellent and 2 were rated as good. All patients achieved bone union after 2 months. Complications occurred in three patients (two transient nerve palsies and one compartment syndrome), and the overall complication rate was 9.7%.

**Conclusions:**

Proximal radioulnar derotational osteotomy followed by plate fixation is a safe and feasible procedure with a low complication rate. The technique can effectively improve the function of the forearm.

**Level of evidence IV:**

Retrospective case series

## Introduction

Congenital radioulnar synostosis (CRUS) is a rare congenital skeletal malformation of the upper limb that can be extremely disabling, especially when it occurs bilaterally or if severe hyperpronation is present [1]. The main feature of this malformation subtype is that the forearm is fixed at the pronation position. The loss of forearm rotation results in hindrance of actions that require this type of motion [2]. The ipsilateral shoulder and wrist canffectively compensate for mild deformity [3]. When pronation deformities are severe, daily activities such as eating, washing, dressing, engaging in personal hygiene care, and accepting objects in the palm of the hand can be severely impaired [4]. Many surgeons have performed proximal radioulnar derotational osteotomy to improve results vary widely due to the small patient groups reported and differences in the duration of follow-up and the surgical techniques selected [3, 5–11].

Many studies have shown that nonsurgical treatments of CRUS are ineffective because of fusion of the proximal ends of the radius and ulna; pronation greater than 60 degrees is indicated for surgical treatment [1, 3, 5, 6]. Historically, surgical separation and reconstruction techniques had theoretically been considered the ideal treatment, taking into account both correction of the deformity and reconstruction of forearm rotational function, but the final outcomes are not satisfactory [12, 13]. Currently, derotational osteotomy remains the most commonly performed procedure in patients with CRUS [3, 5–8], which alters the position of the forearm from hyperpronation to a more functional position to reduce supination limitations and to allow patients to more easily perform activities of daily life. There are many types of osteotomy and fixation methods, including derotational osteotomy at the synostosis site with K-wire fixation [9], one-stage or two-stage double-level derotational osteotomy of the ulna and the radius fixed with plaster casts [7, 10], one- stage double-level derotational osteotomy of the ulna and the radius with K-wire fixation [11, 14], and single osteotomy of the radial diaphysis fixed with plaster casts [8, 15]. The reported complications of the above methods include nerve palsy, compartment syndrome, loss of correction, residual angulation, delayed union, and nonunion.

The purpose of this retrospective study was to assess the proximal radioulnar derotational osteotomy followed by plate fixation for the treatment of CRUS in 31 patients (36 forearms). Based on this relatively large cohort, the outcomes of the surgical correction could be well understood.

## Materials and methods

We treated 62 CRUS patients with surgical methods at our institute from May 2008 until now. Except for 17 cases of the early double-level osteotomy and 1 case of radial head resection, 44 patients (53 forearms) were treated with proximal radioulnar synostosis site derotational osteotomy and plate fixation. Of these patients, 31 (20 boys and 11 girls; 36 forearms) operated from May 2008 to August 2016 met the inclusion criteria and were evaluated. The inclusion criteria were children presenting with either unilateral or bilateral CRUS, skeletally immature children with open growth plates, forearm pronation ≥ 55 degrees, and a score ≤ 10 points based on the Failla [16] classification system. The exclusion criteria were children with inadequate clinical or radiographic data, other combined ipsilateral upper limb anomalies, and a follow-up period of less than 2 years.

In our series, the left elbow was involved in 14 patients, the right elbow was involved in 5 patients, and both elbows were involved in 12 patients (5 patients were operated on both sides, 6 patients on the left side, and 1 patient on the right side). The mean age at the time of surgery was 4.87 ± 3.06 (range, 2 to 13) years. All patients were subjected to X-ray classification based on the shape and position of the radial head according to the Cleary and Omer types [2]. There were no type I cases (fibrous synostosis with a normal-appearing radial head and normal radiocapitellar alignment), 9 (25%) type II cases (bony synostosis with a normal-appearing radial head and normal radiocapitellar alignment), 23 (64%) type III cases (bony synostosis with a hypoplastic and posteriorly dislocated radial head), and 4 (11%) type IV cases (bony synostosis with a hypoplastic and anteriorly dislocated radial head) (Table 1).

**Table 1:** Details of patients (*n* = 31)

Patients	
Side (left/right/bilateral)	14/5/12
Gender (male/female)	20/11
Cleary and Omer types (I/II/III/IV)	0/9/23/4
Age at surgery (mean ± SD; years)	4.87 ± 3.06 (2–13)
Follow-up (mean ± SD; months)	55.19 ± 27.10 (24–123)
Amount of correction (mean ± SD; degrees)	70.86 ± 9.58 (50–90)
The mean union time (months)	2
Complication (patients)	3

### Surgical technique

Under general anesthesia, the patient was placed in the supine position. A tourniquet was applied to the upper arm to obtain a bloodless operating field. A 5-cm longitudinal incision was made over the proximal ulnar ridge. The periosteum was incised and raised to expose the synostosis. A transverse osteotomy was performed through the synostosis, and the forearm was gradually derotated to the target position (20 degrees of supination to 10 degrees of pronation). During surgery, care was taken to avoid injuries to the radial nerve and its branches, especially at the level of the radial neck. In addition, derotational osteotomy is more prone to nerve and blood vessel torsion damage; therefore, the osteotomy must be performed under the periosteum and must be handled gently, and rough peeling must be avoided. The osteotomy site was temporarily stabilized with a 2.0-mm K-wire in an oblique fashion. The osteotomy site was fixed with a Φ2.7-mm locking plate and screw system (Zheng Tian Medical Instrument Co., Ltd. Tianjin, China). Full flexion and extension of the elbow joint were confirmed to exclude any impingement of the joint. Acceptable forearm rotation was confirmed with the shoulder adducted and the elbow flexed at 90 degrees. The wound was closed in layers.

### Postoperative care

After surgery, the elbow was protected using an above-elbow plaster splint for 4 weeks, with the elbow joint at 90 degrees of flexion and the forearm in the neutral position. Close observation for signs of edema and altered peripheral circulation, such as severe pain, numbness, and pale or blue coloring of the affected limb, was performed during the immediate postoperative period, and overtightening of the plaster splint was avoided. The affected limb was lifted on a pillow above the heart for the first three postoperative days. Emergency release accessories were implemented to avoid the occurrence of compartment syndrome. An X-ray of the forearm was acquired 4 weeks after surgery to observe consolidation of the osteotomy. We usually removed the plate and screw system 3–6 months after surgery because the implants may interfere with the growth of the children’s ulna and radius.

### Outcome assessments

Bone union of the osteotomy site was evaluated postoperatively by X-ray, and the incidence of complications was calculated. Evaluation of the surgical effect was first performed by preoperatively and postoperatively measuring the axial position of the forearm to determine improvements of forearm function. The pronation deformity was measured with the patient’s elbow held fixed to the side of the chest and the forearm at 90 degrees of flexion, and the angle between the longitudinal axis of the humerus and the line of the radial and ulnar styloid processes was measured with a goniometer [3, 11]. The forearm function of the children was also assessed via the classification system established by Failla et al. [16] for 15 tasks described by Morrey et al. [17] (Table 2).

**Table 2:** The functional ranges of rotation of the forearm classification system used by Failla et al.

Quantity	Daily activities	Complete:1 point/cannot complete:0 point
1	Touch hand to the vertex (head)	1/0
2	Touch hand to the occiput	1/0
3	Touch hand to the neck	1/0
4	Touch hand to the chest	1/0
5	Touch hand to the waist	1/0
6	Touch hand to the sacrum	1/0
7	Touch hand to the shoe	1/0
8	Pour from a pitcher	1/0
9	Put glass to the mouth	1/0
10	Cut with a knife	1/0
11	Put fork to the mouth	1/0
12	Use a telephone	1/0
13	Read a newspaper	1/0
14	Rise from a chair	1/0
15	Open a door	1/0
	Total score	0–15

Excellent, 15 points; good, 10–14 points; fair, 6–9 points; and poor, < 6 points

### Statistical analysis

Data were analyzed with SPSS Statistics 20.0 (IBM, USA). The Kolmogorov-Smirnov test was applied to analyze the pronation deformity and pre- and postoperative scores. Normal distributions were analyzed with a paired *t* test. Data are expressed as the *x* ± *s*. Differences between pre- and postoperative Failla classification scores were analyzed with the Wilcoxon signed-rank test. A *p* value < 0.05 was considered statistically significant.

## Results

The mean follow-up duration was 55.19 ± 27.10 (range, 24 to 123) months. The mean initial pronation deformity was 62.92 ± 7.11 (range, 55 to 80) degrees, resulting in a mean final position of 7.94 ± 7.25 degrees of supination (range, 20 degrees of supination to 10 degrees of pronation) (*t* = 44.394, *p* < 0.0001) (Table 3). The mean correction achieved was 70.86 ± 9.58 (range, 50 to 90) degrees. There were no significant changes in elbow flexion and extension compared to the preoperative movement. Based on the Failla [16] classification system, the preoperative assessment of the forearms was as follows: 2, good; 30, fair; and 4, poor. At the final follow-up, 34 forearms were excellent and 2 were good, in which the forearm’s position was at 10 degrees of pronation with statistical significance (*Z* = − 7.179, *p* < 0.0001) (Table 4). The rate of excellent or good forearm function increased from 5.6% preoperatively to 100% postoperatively, and the mean score increased significantly from 8.42 ± 1.42 (range, 5 to 10) points preoperatively to 14.89 ± 0.46 (range, 13 to 15) points postoperatively (*t* = − 28.073, *p* < 0.0001) (Table 3). Bone union was achieved in all patients after 2 months. There were no cases of correction loss, infection, or broken plate. Complications occurred in three patients (two cases of transient nerve palsies and one case of compartment syndrome), and the overall complication rate was 9.7%. The patient with compartment syndrome was treated with the release of the flexor digitorum tendon 1 year after surgery and gained good functional recovery of the affected hand. The two cases of transient nerve palsy resolved spontaneously within 2 months.

**Table 3:** Comparison of results of before and after surgery in the operated forearms (*x* ± *s*)

	*n*	Pronation deformity (mean ± SD; degrees)	Score (mean ± SD; points)
Preoperative	36	62.92 ± 7.11	8.42 ± 1.42
Postoperative	36	− 7.94 ± 7.25	14.89 ± 0.46
*t* value		44.394	− 28.073
*p* value		< 0.0001	< 0.0001

**Table 4:** The forearm function by the Failla classification system

	Preoperative	Postoperative
Excellent	0	34
Good	2	2
Fair	30	0
Poor	4	0
*Z* value	− 7.179	
*p* value	< 0.0001	

### Typical case

**Fig. 1:**
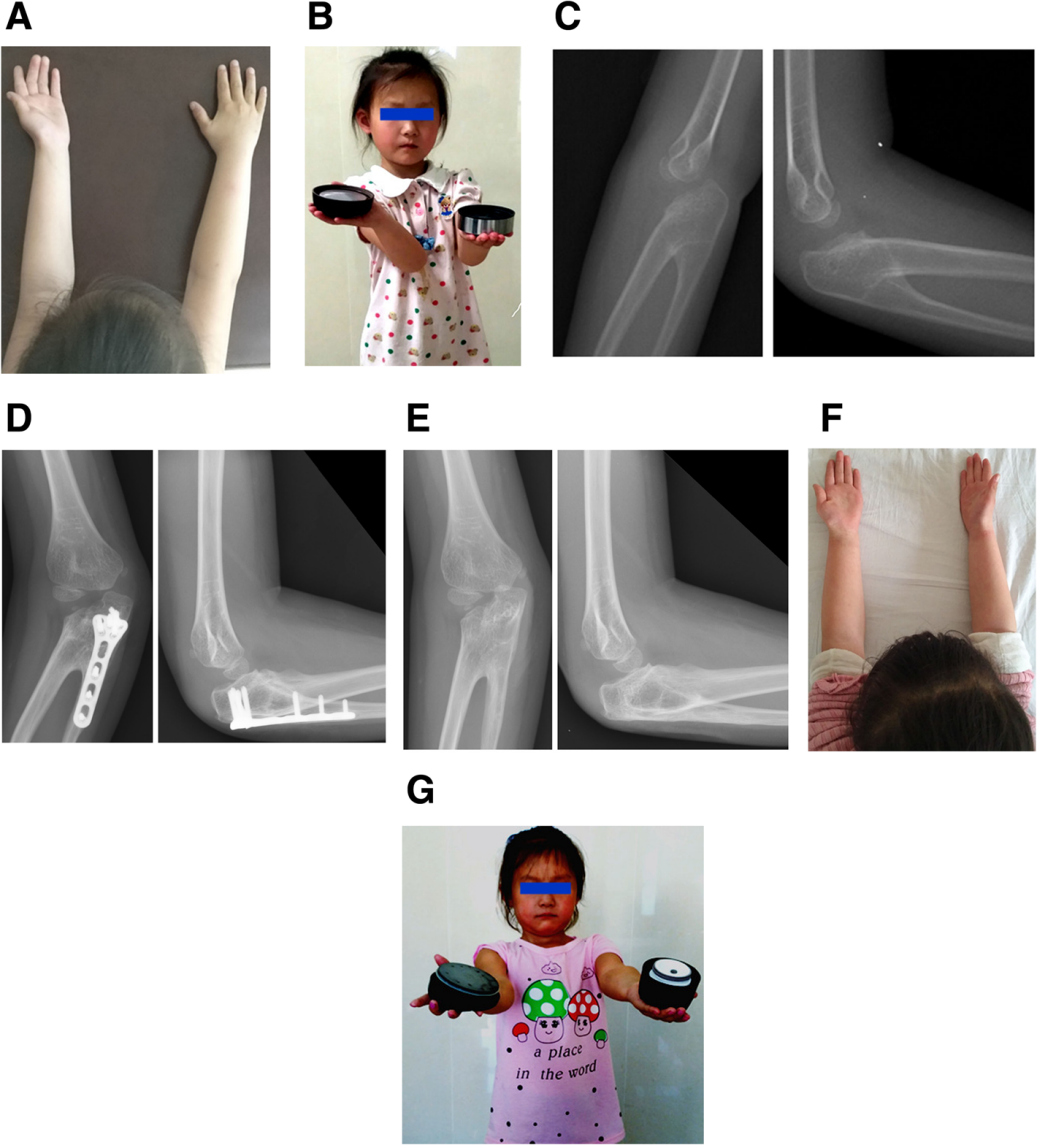
**a** A 6-year-old girl who had proximal radioulnar synostosis in her right forearm that was fixed in the pronation position. **b** She experienced difficulty in daily living activities, such as holding a bowl in the palm, washing, dressing, and engaging in personal hygiene care. Her preoperative Failla score was 7 points. **c** Preoperative anteroposterior and lateral views showing right proximal radioulnar synostosis (type III). **d** Bone union was achieved at the osteotomy site, and the implants were removed 4 months after surgery. **e** X-ray showing good remodeling at the osteotomy site at 2 years after surgery. **f** The rotational function of the right forearm was significantly improved after 2 years. **g** The patient’s daily activities were greatly improved

A 6-year-old girl presented to our department who was described by her parents as having her palm fixed downward and unable to perform supination. She complained of disability in her daily living activities, such as washing, dressing, holding a bowl in the palm, and engaging in personal hygiene care (Fig. 1a, b). Radiological examination revealed that the patient suffered from type III right CRUS (Fig. 1c). A physical examination revealed a fixed pronation deformity of 70 degrees in the right forearm, while extension and flexion of the right elbow joint were not limited. Based on the Failla [16] classification system, only seven tasks could be completed preoperatively. The following tasks could not be performed: touching the vertex (head), chest, neck, waist, sacrum ,or shoe; raising a glass to the mouth; and using a telephone. Derotational osteotomy was performed at the proximal radioulnar fusion site, and the forearm was fixed at 20 degrees of supination with a plate. At 4 months postoperatively, the osteotomy site was healing well, and the plate and screw system was removed (Fig. 1d). At the 2-year follow-up (Fig. 1e), the child’s right forearm was fixed in a position of 20 degrees of supination, without any complications, and the flexion and extension activities of the right elbow were not limited. Her forearm function had significantly improved, including holding a bowl in the palm, and 15 tasks could be completed. The evaluation level improved from fair (preoperative) to excellent (Fig. 1f, g).

## Discussion

CRUS is a rare deformity thought to be caused by a failure of prenatal longitudinal segmentation with persistence of the cartilaginous anlage between the radius and the ulna during the seventh week of embryogenesis [18]. The resultant bridge may be fibrous or bony [19]. Some reports indicate that it has autosomal dominant inheritance [2, 20].

Over time, the specific indications for operative intervention have been debated. Cleary and Omer [2] believe that operative intervention is rarely indicated and their indications for surgery are based more on functional deficits than absolute forearm position. However, Simmons et al. [1] stated that a pronation over 60 degrees was an absolute indication for surgery. Ogino and Hikino [3] suggested that a fixed pronation of 15 to 60 degrees was a relative surgical indication based on the associated functional deficit. Different cultural habits and customs also affect the indications for surgery. Western people use a knife and fork to eat. Eastern people use chopsticks with the dominant hand and hold a bowl with the nondominant hand, which requires excessive supination. Thus, we selected forearm pronation ≥ 55 degrees and a Failla [16] classification system score ≤ 10 points as the surgical indications in this study.

Some reports have been published on the separation of synostosis and interposition of fat or muscle (or some other material), but recurrence of the ankylosis has still been noted [12, 13]. Kanaya et al. [21] reported bony resection of synostosis with free vascularized tissue interposition in seven cases, and all patients had no fusion recurrence and gained forearm rotation at an average of 3.67 years of follow-up. However, the technique of separation and reconstruction surgery has not gained wide acceptance, and derotational osteotomy currently remains the most commonly performed procedure in patients with CRUS [2, 6, 9, 14]. Hung [14] described derotational osteotomy at the shafts of the proximal radius and distal ulna followed by fixation with K-wire and cast immobilization for 34 patients (52 forearms). Twenty-seven patients (78.8%) exhibited good or excellent results, and five patients developed loss of correction. Simcock [9] demonstrated derotational osteotomy at the synostosis site followed by fixation with crossing K-wires in 26 patients (31 forearms), resulting in a mean final position of 8 degrees of pronation. In that series, complications included 1 case of symptomatic muscle herniation and 3 cases of transient nerve paralysis, 2 of which corresponded to transient anterior interosseous nerve palsies with rotational corrections that exceeded 80 degrees. The other osteotomy approach is at the radial diaphysis and is fixed only with a cast, which has yielded good results and few complications, but this procedure requires a cast change 2 weeks after surgery [15]. In our series, all patients underwent derotational osteotomy at the synostosis site followed by the plate for rigid internal fixation in case of correction loss, and plaster splint was used for external immobilization, which enabled convenient close observation to monitor edema and peripheral circulation as well as ease of release. There were no increases in the incidence of complications by using plate fixation in our study compared with other fixation methods such as K-wires and/or casts reported by other authors [7, 9, 14, 22].

Absolute agreement about the optimal position for the forearm after derotation is also lacking. Several surgeons have advocated 0 to 20 degrees of supination in the nondominant forearm and 0 to 20 degrees of pronation in the dominant forearm [3, 23, 24]. Green et al. [6] suggested that in bilateral cases, the best positions are 30 to 45 degrees of pronation in the dominant forearm and 20 to 35 degrees of supination in the nondominant forearm. Green et al. [6] found that if one forearm is placed in the supinated position, it complements the other at 30 to 45 degrees of pronation to ensure that the patient is able to easily perform tasks requiring supination and pronation; therefore, they suggested that the ideal position is 10 to 20 degrees of supination in unilateral cases.

Our study showed that in 0 (neutral) to 20 degrees of supination, both the dominant and nondominant forearms, had the best function, i.e., the highest score of 15 points based on the Failla [16] classification system. Patients with forearms in this position could perform movements that require forearm pronation, such as using a computer mouse and keyboard and touching the back of the head, and they could also complete movements that require forearm supination, such as holding a bowl in the palm, washing, touching the waist with the palm of the hand, and engaging in personal hygiene care. In our series, 2 children (2 forearms) were unable to touch the waist and the sacrum with corrected forearm positions of 10 degrees of pronation. They had scores of 13 points based on the Failla [16] classification system.

Many investigators have reported major complications of derotational osteotomy, which include transient nerve palsy, loss of correction, residual angulation at the osteotomy site, delayed union, and compartment syndrome [7, 14, 15, 22, 24]. Compartment syndrome is a complication that requires specific precautions [9, 14, 22]. One case of compartment syndrome occurred in our series: the patient was 2 years and 6 months old at the time of the correction surgery and achieved 70 degrees of correction during surgery; this patient received a nerve block with a long-acting anesthetic near the end of surgery for perioperative analgesia and did not complain of pain for almost 11 h after surgery, which resulted in the residents and nurses not closely monitoring forearm edema and the peripheral circulation and missing the opportunity for timely treatment of this complication. Transient nerve palsy may be caused by traction during exposure or correction exceeding 80 degrees, but the condition resolves spontaneously within 3–4 months and does not require treatment [9]. Murase [24] reported a 20-degree loss of correction in 1 of 4 cases, and Hung [14] reported 15–20 degrees of loss of correction in 5 of 52 forearms with derotational osteotomy of the ulnar and radial shaft fixed with K-wire and cast immobilization. There are also studies of derotational osteotomy with only cast immobilization without internal fixation [7, 8, 10, 15]. In these series, the rotation of the forearm is determined only by the cast; therefore, the surgeons must be cautious regarding the position of the forearm in the cast and may change the patient’s casts until bone fusion is achieved. In this study, we fixed the osteotomy of the synostosis site with plate and plaster splint immobilization, and no cases of correction loss, residual angulation, delayed union, nonunion, and broken plate occurred among the 36 forearms from age 2 to 13 years. The disadvantages are an extensive exposure and a secondary surgery to remove the implants.

The study has limitations that are inherent to a retrospective study; therefore, its results must be interpreted with caution and need to be confirmed in a larger patient population.

## Conclusions

Proximal radioulnar derotational osteotomy followed by plate fixation is a safe and feasible technique in patients with CRUS. The technique has a low complication rate and can effectively improve the function of the forearm and the quality of the patient’s life.

## Abbreviation

CRUS: Congenital radioulnar synostosis
